# Myofiber stearoyl-CoA desaturase 1 moderates MAFLD and metabolic syndrome in obese mice

**DOI:** 10.3389/fphys.2026.1826756

**Published:** 2026-08-03

**Authors:** Andrew Guilfoyle-Speese, Cody Bridgewater, Caleb Padgett, Hunter Sellars, James Mintz, David Fulton, David Stepp

**Affiliations:** 1 Vascular Biology Center, Medical College of Georgia, Augusta University, Augusta, GA, United States; 2 Department of Pharmacology and Toxicology, Medical College of Georgia, Augusta University, Augusta, GA, United States; 3 Department of Physiology, Medical College of Georgia, Augusta University, Augusta, GA, United States

**Keywords:** diabetes, hepatosteatosis, myosteatosis, obesity, steatosis

## Abstract

**Introduction:**

Obesity-induced ectopic lipid accumulation inside skeletal muscle (SKM) (myosteatosis) and the liver (hepatosteatosis) contributes to local metabolic dysfunction and serves as a launchpad for the progression of diabetes. While stearoyl-CoA desaturase 1 (SCD1), an ER-bound enzyme that catalyzes the rate-limiting step in monounsaturated fatty acid (MUFA) synthesis, is widely recognized as a lynchpin facilitator of steatosis, much controversy exists on its efficacy as a therapeutic target. In particular, a total lack of SKM-specific SCD1 KO models has greatly limited our understanding of SCD1-mediated myosteatosis’ relative contribution to metabolic syndrome.

**Methods:**

Global SCD1 knockout mice bred on the leptin receptor mutant (db/db) background (db/SCD1 mice) and SKM-specific inducible ACTA1-cre mice bred on the SCD1^fl/fl^ background overexpressing agouti-related peptide (AgRP) had their body/tissue composition analyzed via nuclear magnetic resonance (NMR) spectrometry. Insulin-mediated metabolism was analyzed by screening blood/plasma for related metabolites. Lastly, we performed RT-qPCR to screen target metabolic genes.

**Results:**

Global SCD1 deletion in db/db mice reduced weight gain and steatosis, leading to pathological liver remodeling and severe diabetes. Next, we found that SKM-restricted SCD1 deletion in adult mice with established obesity increased AgRP-induced weight gain while preserving whole-body fat composition. SKM SCD1 KO led to reduced myosteatosis while exacerbating hepatosteatosis and indices of insulin insensitivity. Whereas SKM responded to lack of myocellular SCD1 by inhibiting expression of trans-endothelial fatty acid chaperone FABP4, hepatosteatosis coincided with upregulation of SCD1 and downregulation of master regulator of fatty acid oxidation (FAO) PPARα in the liver.

**Conclusions:**

Loss of peripheral leptin-mediated FAO and SCD1-regulated lipid storage act synergistically to exacerbate hyperlipidemia, hyperglycemia, and multi-organ quality decline, collectively promoting systemic inflammation and diabetes progression. Interestingly, AgRP mice adapt to selective inhibition of SCD1-mediated myosteatosis by expanding hepatosteatosis and precipitating metabolic syndrome. Gene expression data imply SCD1-KO–mediated impaired SKM lipid flux inhibits liver lipid metabolism in favor of storage. These results indicate a potent link between intramyocellular lipid storage capacity and the progression of metabolic-associated fatty liver disease.

## Introduction

Obesity represents a global healthcare crisis accounting for approximately 4–6 million deaths annually driven by cardiovascular disease, cancer, diabetes, and kidney disease (Bluher, 2019; Okunogbe et al., 2021). Obesity is a primary contributor to type 2 diabetes (T2DM) and metabolic-associated fatty liver disease (MAFLD), both of which are characterized by visceral fat accumulation, chronic inflammation, and insulin resistance (Fabbrini et al., 2010; Liu et al., 2020). Mechanisms linking these processes to end-stage disease remain incompletely understood, and crosstalk between metabolic compartments remains poorly defined.

An early-onset hallmark of obesity is the ectopic deposition of fat into non-adipose tissues, particularly the liver (hepatosteatosis) and skeletal muscle (SKM) (myosteatosis) (Snel et al., 2012). These lipids are primarily stored as stable and relatively nonreactive triacylglycerides (TAGs) and esterified cholesterol; however, as supply supersedes storage and oxidative-disposal capacities, highly reactive lipid intermediaries may accumulate and act as instigators of mitochondrial and ER stress (Prasun et al., 2021). This lipotoxicity contributes to the progression of local inflammation and insulin resistance that drive metabolic syndrome (Britton and Fox, 2011; Janssen, 2024).

Stearoyl-CoA desaturase 1 (SCD1) is the rate-limiting enzyme in TAG and cholesterol-ester synthesis. SCD1 is a microsomal enzyme that catalyzes the conversion of saturated fatty acids (SFAs) into monounsaturated fatty acids (MUFAs) (Miyazaki and Ntambi, 2003). Elevated expression of this protein has been correlated with obesity, diabetes, metabolic dysfunction, and steatosis severity (Dobrzyn and Ntambi, 2004; Popeijus et al., 2008; Sampath and Ntambi, 2011). In both humans and mice, SCD1 is the principal SCD isoform expressed in the liver, adipose, skin (sebaceous glands), and SKM, among which the liver and adipose represent the focus of the vast majority of SCD1 research (Miyazaki and Ntambi, 2003; AM et al., 2017). In obese humans, SKM SCD1 expression and activity show strong positive correlations to BMI, plasma insulin, and TG synthesis while being negatively correlated to fatty acid oxidation (FAO) (Hulver et al., 2005). Additionally, global and tissue-restricted (liver, adipocyte, and skin) SCD1 KO and rescue studies using mouse and rat obesity models have characterized the functions of SCD1 in these tissues (Jiang et al., 2005; MacDonald et al., 2009; Sampath et al., 2009; Liu et al., 2011; Aljohani et al., 2019). However, the role of SKM SCD1 activity in obesity is poorly understood and far more controversial, with both deleterious and protective roles speculated (Stamatikos and Paton, 2013). This lack of consensus likely reflects an absence of *in-vivo* obesity models characterizing SKM-specific SCD1 inhibition/KO.

In this study, we use multiple novel mouse models to determine the role(s) of SCD1-mediated steatosis in the pathophysiology of obesity. Specifically, using global SCD1 KO mice bred on the *db/db* background, we examine the effects of whole-body SCD1 KO in a model of profound hyperphagia and metabolic dysfunction. Next, to determine the role of SCD1 in SKM, we deploy a novel model of obesity using brain-targeted adenoviral delivery of hyperphagic AgRP on cre/flox backgrounds, allowing us to delete SCD1 in the muscle of obese mice and determine its function in established obesity. Taken together, these studies provide the first critically tested description of myosteatosis’ purpose in the context of obesity.

## Materials and methods

### Animals and animal maintenance

C57BL/6J mice heterozygous for mutation in the leptin receptor (Jackson Labs; strain no. 000697) were crossed with mice lacking SCD1 (Jackson Labs; strain no. 006201) to generate db^+/±^/SCD1^+/±^: H_db_H_SCD1_ (lean), H_db_K _SCD1_ (lean SCD1 KO), K_db_H _SCD1_ (obese), and K_db_K _SCD1_ (obese SCD1 KO) genotypes. The nomenclature of the global colony of mice is that H denotes heterozygous expression and K denotes absence, either by deletion of the mutation of the subscripted gene. Heterozygous mice were used as a control as an assumption of parsimony that a difference detected with one gene difference would be more conservative than two. Inducible SKM SCD1 KO mice were generated by crossing mice expressing ACTA1-MerCreMer (Jackson Labs; strain no. 031934) with SCD1*fl/fl* (ACTA1-SCD1*fl/*^±^) mice generously provided by the Ntambi lab from Madison, Wisconsin (available at Jackson Labs; strain no. 041157). Following AgRP AAV-induced hyperphagia, 35-week-old mice (when mice were >50 g) were treated with tamoxifen (80 mg/kg, ~4 mg/50 g mouse) via IP injection of tamoxifen/corn oil solution (0.032 mg/µl, 0.125 ml/50 g mouse) once per day over five days, which was then repeated for an additional five days after a two-day break. Controls for inducible SKM SCD1 KO were ACTA-MerCreMer treated with tamoxifen. ACTA1-SCD1*fl/*^±^ mice that did not achieve >50 g of body mass by 35 weeks of age or fully recover from tamoxifen treatments were excluded from further experiments. Obese mice were used in these studies, as lean mice on either background display no steatosis in any tissue. All experiments involving animals were conducted under Institutional Animal Care and Use Committee (IACUC) approval and in accordance with the NIH Guide for the Care and Use of Laboratory Animals. Furthermore, due to challenges in breeding K_db_K_SCD1_ mice as well as the timespan to establish the ACTA1-SCD1*fl/*^±^ colony and to achieve sufficient AgRP-induced body mass, the minimal number of biological replicates of each (*n* ≥ 6 and *n* = 4–5, respectively) was used for *in/ex-vivo* whole-body and tissue/organ characterization. db^+/±^/SCD1^+/±^ and ACTA1-SCD1*fl/*^±^ male mice were euthanized at approximately 24 and 40 weeks of age, respectively, by anesthetic overdose (soflurane, 5%) with decapitation as a secondary method. A list of mouse genotyping primer sequences is provided in **Supplementary Table 1**.

### AAV production and administration

Human AgRP (AAV-CapB10-Syn1-AGRP, titer: 2.14 × 10^13^ GC/ml, Lot# 4A703d2) was purchased from AAVnerGene Inc. Previously, CAG-NLS-GFP AAV.PHP.eB control AAVs were obtained from Addgene (CAT#: 104061-PHP.eB) and used to verify brain specificity (Sellers et al., 2026). SKM SCD1 KO, Obesity was induced via a single 50 µl retro-orbital injection of AAV (1 × 10^11 gc/mouse) in 8-week-old ACTA1- SCD1*fl/*^±^ mice while under isoflurane anesthesia, as described previously *(*Sellers et al., 2026*).*

### Metabolic phenotyping

Mouse body content was determined via nuclear magnetic resonance (NMR) using a Minispec Body Composition Analyzer (Bruker; model no. LF90II) to determine whole-body fat and lean percentages immediately prior to sacrifice. Tissues were isolated, and liver and gastrocnemius tissues were also subjected to NMR to determine intra-organ fat versus lean content. Mice were fasted for 4h before euthanasia, and blood was collected by heparinized syringe. Blood samples were analyzed for fasting blood glucose using a standard glucometer (AlphaTrak, Kalamazoo, MI, USA) and for HbA1c using a multi-test A1CNow HbA1c system (PTS Diagnostics). Plasma was separated via centrifugation. A blinded technician analyzed plasma samples for total cholesterol (FujiFilm), insulin (Alpco), free fatty acids (Sigma), triacylglycerols (FujiFilm), thiobarbituric acid reactive substances (Cayman), TNFα (BioLegend), aspartate aminotransferase (Sigma), and alanine aminotransferase (Abcam). Additionally, a cohort of mice (*n* = 3–4/genotype) was monitored by a blinded technician using Comprehensive Lab Animal Monitoring System (CLAMS) cages (Columbus Instruments) to measure food and water intake, energy expenditure, respiration, and activity. Mice were allowed to acclimate to the CLAMS for 24h, then monitored for 3 continuous days (i.e., three technical replicates/mouse) for the purpose of data collection.

### Histology and analysis

All sample paraffinization, slide preparation, and staining were performed by our dedicated Electron Microscopy and Histology Core Facility at Augusta University (RRID: SCR_026810). Hematoxylin & Eosin (H&E), Masons Trichome, and Oil Red O staining were carried out in accordance with Thermo Scientific guidelines (https://documents.thermofisher.com/TFS-Assets/LSG/manuals/IS87019%20GramStainTissuChromaview%20IFU.pdf, [[NoYear]]). For immunohistochemistry (IHC), frozen slides were air-dried for 15 min at room temperature and then fixed in 4% formaldehyde for 10 min at room temperature. After fixation, the slides were washed in PBS three times and incubated in blocking solution (1% BSA, 2.5% horse serum, and 0.5% Triton X-100) for 1h at room temperature. Slides were then incubated overnight at 4 °C with the primary antibody (mouse anti-laminin, Novus, NB600-883, or 1:500; mouse anti-SCD1, Invitrogen, PA5-95762, 1:250). The following day, the slides were washed 3 times with PBS and incubated for 2h at room temperature with the fluorescent secondary antibody (Texas Red anti-mouse IgG, Vector, TI2000, 1:250). Laminin was co-stained with autoDOT MDH (Abcepta, SM1000a, 1:500), a fluorophore-labeled dye that preferentially segregates into neutral lipid droplets (LDs), in order to visualize differences in intramyocellular lipid accumulation. Finally, the slides were washed three times with PBS and coverslipped using Fluoroshield (Sigma, F6182). Images were captured using a fluorescence microscope. Median myofiber cross-sectional area (CSA) was determined semi-autonomously via cellular segmentation and analysis of H&E-stained whole-gastrocnemius muscles with Cellpose-SAM and Fiji, respectively, as described (Marks et al., 2025). Using their standard/preset settings, longitudinal, damaged, and out-of-focus myofibers as well as non-myofiber structures were automatically excluded resulting in >4,000 myofibers analyzed/sample. From the initial four ACTA1-SCD1*fl/*^−^ samples, one was excluded from histological analyses due to pervasive freeze-thaw artifacts.

### Gene expression analysis

To assess gene expression, RNA was isolated via phase separation using TRIzol-chloroform. cDNA was synthesized using All-In-One 5× RT MasterMix (ABM, CAT# G592). qPCR was performed using BlasTaq™ 2× qPCR MasterMix (ABM, CAT# G891) in a CFG-Connect Real-Time PCR Detection System (Bio-Rad). Expression fold change was calculated using the 2-ΔΔCT method and normalized to either 18s or GAPDH as an internal control. List of mouse primer sequences provided in **Supplementary Table 1**.

### Protein analysis

Frozen muscle tissue was crushed and suspended in Laemmli (1X) buffer and boiled at 95 °C for 5 min. Samples were run on a Mini-PROTEAN TGX precast gel (Bio-Rad) for 2h at 100V. The gel was then placed in a cassette stacked with a nitrocellulose membrane and soaked with transfer buffer (100 ml Transfer Solution (144 g Glycine, 30.2 g Tris-Base to 1,000 L), 200 ml 100% methanol, 700 ml DI H2O) and then loaded into a Trans-Blot Turbo transfer system (Bio-Rad). The transfer was performed for 6 min at 2.5A_25V. The membrane was washed for 5 min, 2 times with DI water, then soaked in Ponceau for 15 min, and then imaged for total protein with a ChemiDoc imaging system (Bio-Rad). The membrane was then washed repeatedly with DI water until it ran clear and then blocked in 5% milk for 1h and washed for 15 min, 3 times with 1× TBST. The membrane was then cut to separate the desired proteins for probing. Membrane slices were probed with their respective antibody (**Supplementary Table 1**) overnight at a dilution of 1:1000 in 5% milk. The membrane was then washed again as above and probed with secondary antibody at a dilution of 1:2500 in 5% milk and incubated at 25 °C for 1h, then washed again as previously described. ECL was used to activate the secondary antibodies per the manufacturer’s instructions. The strips were lined up accordingly to appear prior to cutting, then gently dabbed with Kim-wipes to remove any excess ECL. Activated membranes were then imaged via chemiluminescence using the ChemiDoc imaging system. Samples were analyzed via Fiji and normalized to total protein.

### Statistical analyses

A Student’s t-test and a one-way analysis of variance (ANOVA) were calculated to examine differences between two or four groups, respectively. For data sets that did not meet criteria for normality and equal variance, or for sets with *n* < 5, the exact versions of non-parametric tests were used as they are appropriate tests to use with smaller sample sizes. Mann–Whitney tests were calculated to examine differences between two groups, Dunnett’s multiple comparisons tests were calculated to examine differences between Wt control and SCD1^fl/±^ groups, and Tukey’s tests were calculated to examine differences among four groups. All analyses were performed with GraphPad Prism 10.1 (GraphPad Software Inc.). Data are expressed as mean ± standard error of the mean. *P* ≤ 0.05 was used as the minimum criterion for significance with specific values denoted in the legends of each figure.

## Results

### Whole-body SCD1 KO limits obesity-Driven weight gain and body composition changes without affecting activity or food consumption

**Figure 1:**
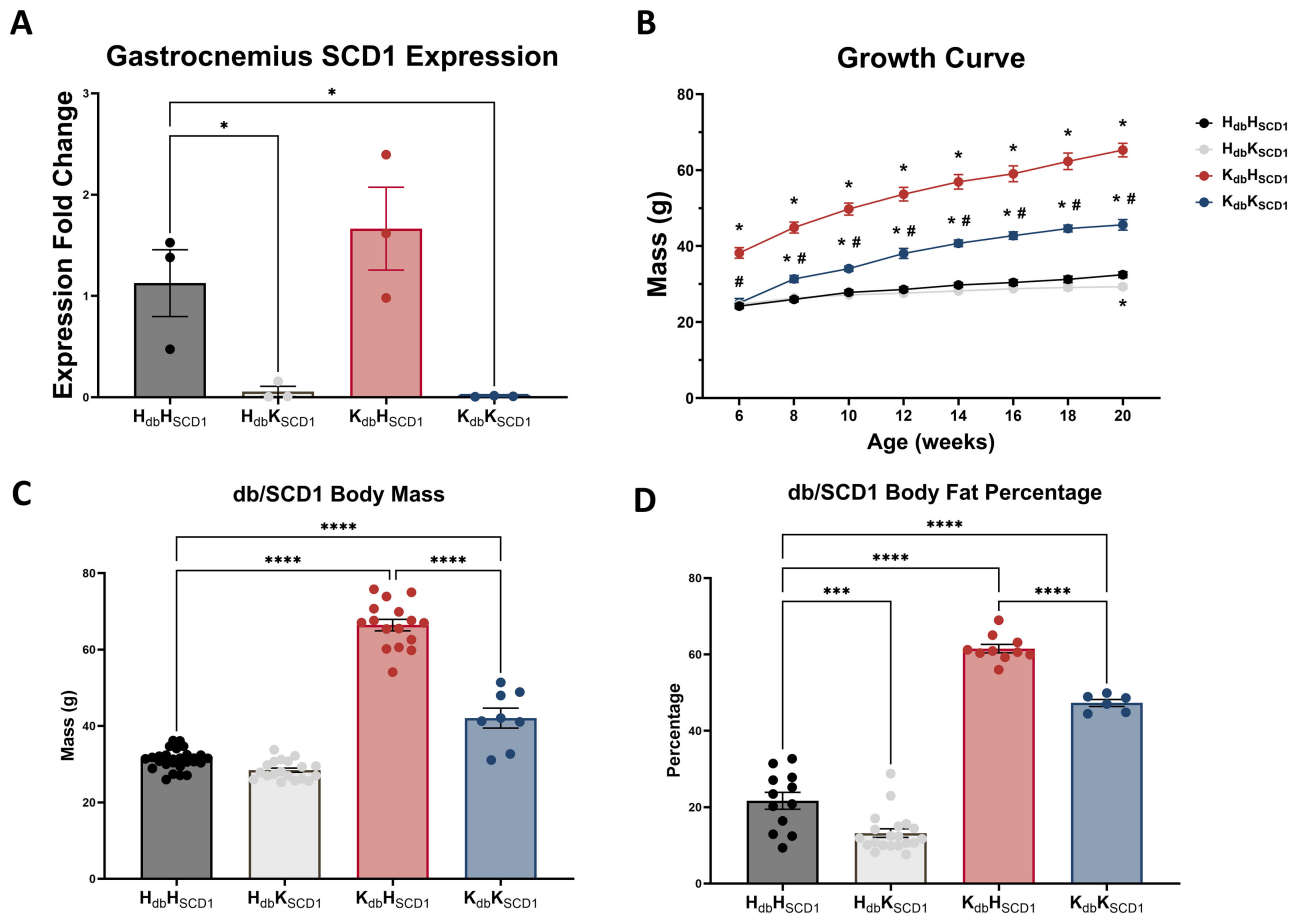
Global SCD1 KO significantly attenuates obesity-driven changes to whole-body mass and composition. **(A)** RT-qPCR–determined gastrocnemius SCD1 mRNA levels [analysis of variance (ANOVA) and Fisher’s LSD, *n* = 3]. **(B)** Mouse body mass growth curve (mixed effects analysis, *n* ≥ 3) and **(C)** (ANOVA and Tukey, *n* ≥ 8) were used by Minispec Body Composition Analyzer to calculate. **(D)** Nuclear magnetic resonance (NMR)–determined body fat percentages (ANOVA and Tukey, *n* ≥ 6). All data represented as mean ± SEM [*P < 0.05, **P < 0.01, ***P < 0.001, ****P < 0.0001; in panel **(B)** *P < 0.05 vs. H_db_H_SCD1_, #*P* < 0.05 vs. K_db_H_SCD1_.

As reported previously (Butcher et al., 2017; Butcher et al., 2018; Larion et al., 2024), obesity on a db/db background is associated with marked increases in body mass, hyperlipidemia, loss of glycemic control, renal hypertrophy, adrenomegaly, and gonadal hypertrophy (**Table 1A**). SCD1 KO (K_SCD1_) was confirmed via RT-qPCR and resulted in significantly attenuated weight gain in obese (LEPR knockout, K_db_) mice by ~24 g at 20 weeks of age (**Figures 1A–C**). Obese_SCD1 double-KO (K_db_K_SCD1_) animals displayed a 23% reduction in whole-body fat content percentage (**Figure 1D**). Additionally, we found that SCD1 KO results in marked elevations in all CLAMS-determined indices of metabolic activity/energy expenditure in lean (“H”eterozygous for db_SCD1 KO, H_db_K_SCD1_) mice that were absent in obese mice (**Table 2**). 

**Table 1:** db/SCD1 physiologic and metabolic phenotype data.

A.	Organ mass			
Genotype:	H_db_H_SCD1_	H_db_K_SCD1_	K_db_H_SCD1_	K_db_K_SCD1_
Heart (mg)	162.2 ± 4.9	157.3 ± 5.8	152.8 ± 3.5	147.6 ± 5.4
Kidney (mg)	205.8 ± 5.1	211.5 ± 10.8	255.5 ± 8.8^ab^	243.4 ± 9.8^a^
Gonad (mg)	107.4 ± 1.3	92.74 ± 3.9^a^	86.73 ± 4.9^a^	65.91 ± 6.8^ab^
Adrenal (mg)	2.38 ± 0.1	2.51 ± 0.2	3.78 ± 0.2^ab^	4.46 ± 0.6^ab^
B.	Blood/plasma analysis			
Genotype:	H_db_H_SCD1_	H_db_K_SCD1_	K_db_H_SCD1_	K_db_K_SCD1_
Insulin (ng/ml)	0.54 ± 0.1	0.36 ± 0.1	4.03 ± 0.7^ab^	2.85 ± 0.8^ab^
Leptin (pg/ml)	861.5 ± 148.5	120.4 ± 49.6^a^	2801.9 ± 100.0^ab^	2541.4 ± 68.9^ab^
Triglyceride (mg/dl)	29.88 ± 3.3	29.35 ± 4.9	80.9 ± 5.3^ab^	97.64 ± 7.8^ab^
Cholesterol (mg/dl)	83.33 ± 6.6	76.31 ± 12.4	157.32 ± 11.3^ab^	176.9 ± 5.0^ab^
NEFA (ng/ml)	1.65 ± 0.07	1.48 ± 0.07	2.94 ± 0.05^ab^	3.12 ± 0.23^ab^
Glucose (mg/dl)	204.2 ± 10.9	206.7 ± 13.2	361.5 ± 40.3^ab^	610.9 ± 34.2^abc^
TBARS (µM)	0.20 ± 0.016	0.18 ± 0.016	0.57 ± 0.021^ab^	0.50 ± 0.044^ab^
HbA1c (%)	4.48 ± 0.046	4.23 ± 0.069^a^	6.35 ± 0.315^ab^	8.58 ± 0.485^abc^
TNFα (pg/ml)	24.64 ± 0.8	24.84 ± 0.6	50.71 ± 3.2^ab^	85.49 ± 14.6^abc^
AST (U/L)	31.928 ± 1.57	47.165 ± 2.67^a^	85.549 ± 4.02^ab^	120.58 ± 1.19^abc^
ALT (pg/ml)	29.931 ± 0.41	33.772 ± 1.01	56.022 ± 2.75^ab^	86.421 ± 5.16^abc^

Data was analyzed with 1-way ANOVA and Tukey’s multiple comparisons tests. All data represented as mean ± SEM (A) N ≥ 6; (B) N ≥ 3, a = P < 0.05 versus H_db_H_SCD1_, b = P < 0.05 versus H_db_K_SCD1_, c = P < 0.05 versus K_db_H_SCD1_.

**Table 2:** Comprehensive lab animal monitoring system (CLAMS) data (24h avg).

Genotype:	H_db_H_SCD1_	H_db_K_SCD1_	K_db_H_SCD1_	K_db_K_SCD1_
VO2 (mL/kg/h)	3070.5 ± 80.2	5353.3 ± 316^a^	1978.3 ± 73.5^ab^	2666.5 ± 286^bc^
VCO2(mg)	2505.5 ± 59.7	4673.5 ± 291^a^	1685.5 ± 49.4^ab^	2161.3 ± 235^ab^
RER (VCO2/VO2)	0.813 ± 0.23	0.864 ± 0.008	0.847 ± 0.015	0.796 ± 0.026^b^
Heat (kcal/h)	0.474 ± 0.011	0.754 ± 0.018^a^	0.605 ± 0.019^a^	0.556 ± 0.055
Food consumption (g)	3.43 ± 0.44	5.92 ± 0.31	3.93 ± 0.54	3.45 ± 1.13
Water con (ml)	2.93 ± 0.41	6.32 ± 0.55^a^	3.62 ± 0.38	4.68 ± 1.64
Wheel rotations	466.9 ± 222.7	1453.2 ± 544^a^	25.9 ± 11.4^b^	32.3 ± 7.93^b^

Results are daily averages across three technical replicates (i.e., three days). Data was analyzed with 1-way ANOVA and Tukey’s multiple comparisons tests. All data represented as mean ± SEM (N ≥ 3), a = P < 0.05 versus H_db_H_SCD1_, b = P < 0.05 versus H_db_K_SCD1_, c = P < 0.05 versus K_db_H_SCD1_.

### Global SCD1 deletion results in profound sarcopenia and reduced myosteatosis

**Figure 2:**
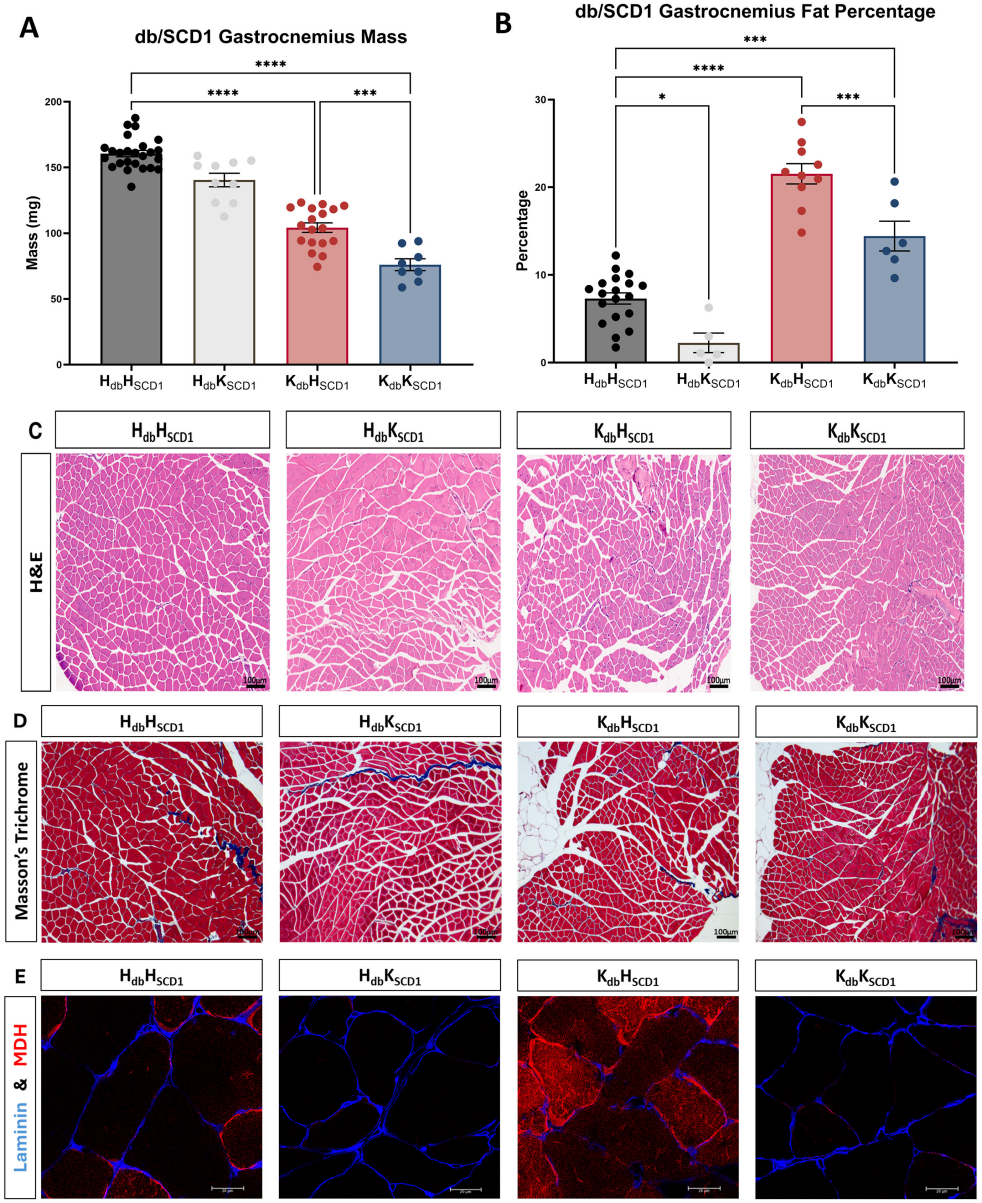
SCD1 KO in obese mice significantly reduces gastrocnemius mass and steatosis. **(A)** Mouse gastrocnemius masses [analysis of variance (ANOVA) and Tukey, *n* ≥ 8] were used by Minispec Body Composition Analyzer to calculate **(B)** nuclear magnetic resonance (NMR)–determined tissue fat percentages (ANOVA and Tukey, *n* ≥ 5). **(C)** Representative H&E (scale bar = 100 µm), **(D)** Mason’s trichome (scale bar = 100 µm), and **(E)** Monodansylpentane (MDH, red) and anti-Laminin (blue) (scale bar = 20 µm) staining of mouse gastrocnemius cross-sections were performed to assess gross morphology and relative intramyocellular lipid content. All data represented as mean ± SEM (*P < 0.05, **P < 0.01, ***P < 0.001, ****P < 0.0001).

To assess the role of SCD1 in the muscle of obese mice, macroscopic and microscopic parameters were evaluated. Obesity-driven sarcopenic 35% loss in gastrocnemius mass was exacerbated to 53% in double KO mice (**Figure 2A**). Furthermore, SCD1 KO attenuated obesity-induced attenuated the increase in gastrocnemius fat content, as assessed by NMR (**Figure 2B**). Next, using hematoxylin and eosin (H&E) and Masons trichrome (MT) staining, we observed that the microscopic architecture of K_db_H_SCD1_ and K_db_K_SCD1_ muscles displayed normal SKM morphology (**Figures 2C, D**). Using Monodansylpentane (MDH) staining, SKM from obese mice displayed marked intracellular lipid accumulation, supporting observations obtained by NMR. Critically, SCD1 deletion resulted in the reversal of intramyocellular MDH+ lipid accumulation in both lean and obese animals (**Figure 2E**). These novel observations reveal that SCD1 is the key regulatory enzyme determining the extent of myosteatosis in the db/db mouse model of obesity.

### SCD1 KO restricts liver hypertrophy and steatosis

**Figure 3:**
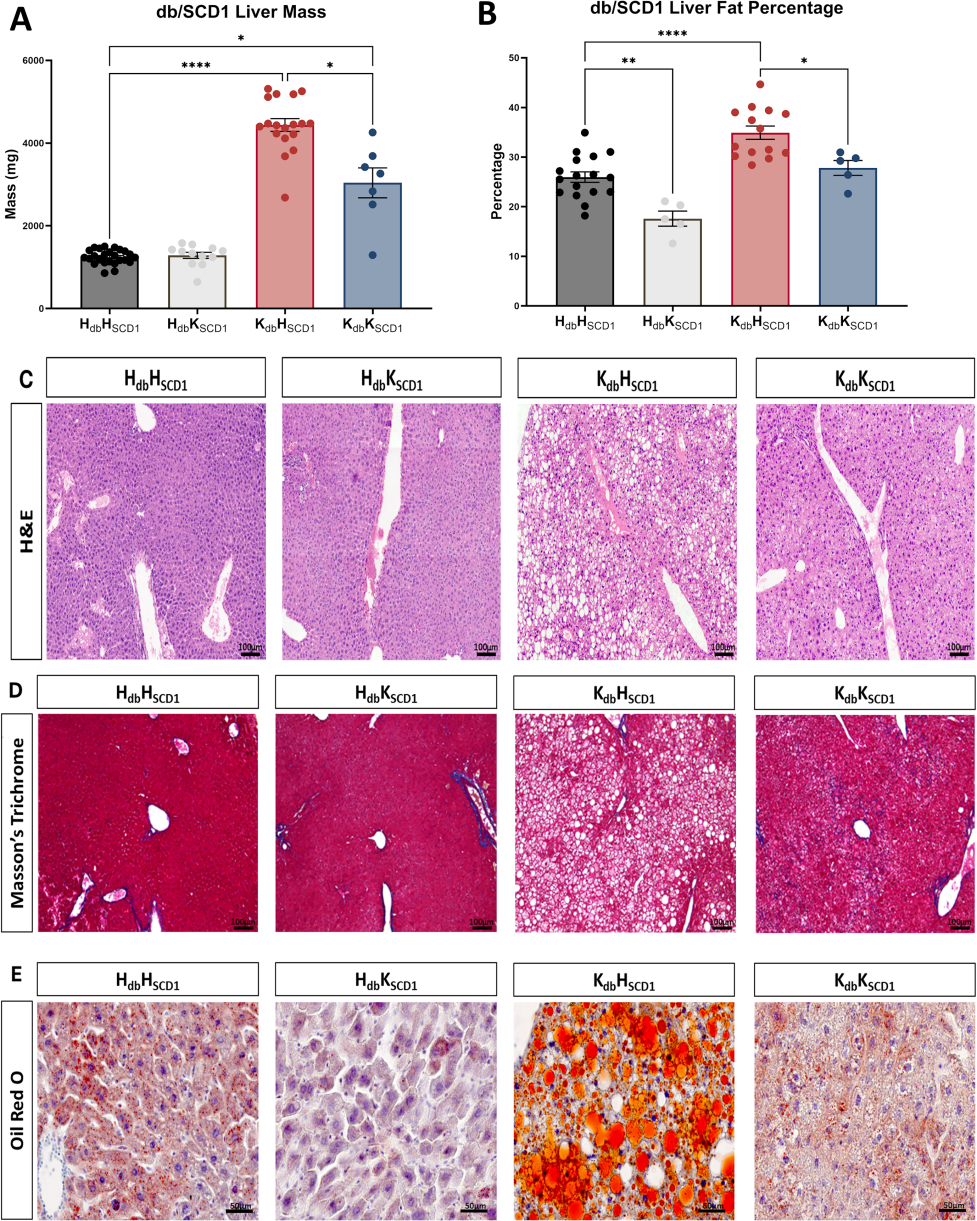
SCD1 KO in obese mice significantly reduces liver mass and steatosis. **(A)** Mouse liver masses [analysis of variance (ANOVA) and Tukey, *n* ≥ 7] were used by Minispec Body Composition Analyzer to calculate **(B)** nuclear magnetic resonance (NMR)–determined tissue fat percentages (ANOVA and Tukey, *n* ≥ 5). **(C)** Representative H&E (scale bar = 100 µm), **(D)** Mason’s trichome (scale bar = 100 µm), and **(E)** Oil Red O (ORO) (scale bar = 50 µm) staining of mouse liver cross-sections were performed to assess gross morphology and relative hepatocyte lipid content. All data represented as mean ± SEM (*P < 0.05, **P < 0.01, ***P < 0.001, ****P < 0.0001).

Similar to SKM, SCD1 KO resulted in profound liver phenotypic changes. As shown in **Figure 3A**, db/db mice showed marked hepatomegaly as documented previously (Butcher et al., 2017; Butcher et al., 2018; Larion et al., 2024). SCD1 deletion limited obesity-mediated gross liver expansion by ~32% and prevented an obesity-induced 34% increase in hepatic fat content, as determined by NMR (**Figures 3A, B**). These data are reinforced by H&E, MT, and Oil Red O staining depicting pathological macrovesicular lipid droplet accumulation and resulting hepatocyte ballooning in obese mice, which are largely prevented by global deletion of SCD1 (**Figures 3C–E**). These observations reinforce findings from previous reports that characterized SCD1 as a gatekeeping enzyme on hepatic steatosis (Aljohani et al., 2019).

### SCD1 KO worsens diabetes in db/db mice

An assessment of the pathological consequences of obesity and SCD1 deletion is shown in **Table 1B**. SCD1 KO significantly lowered plasma leptin by 84% in lean but not obese mice, likely due to reduced fat mass. Furthermore, the approximately threefold to fourfold increase in insulin and leptin concentrations in db/db mice coincided with hyperlipidemia (approximately 2-fold increase in plasma triglyceride (TG), cholesterol, and non-esterified fatty acid (NEFA) concentrations) and hyperglycemia (1.6-fold and 2-fold increases in blood glucose and HbA1c, respectively), the latter of which was significantly worsened (threefold and twofold increases in blood glucose and HbA1c, respectively) in K_db_K_SCD1_ mice. The presence of metabolic stress was further confirmed in db mice by a ≥2-fold increase of thiobarbituric acid reactive species (TBARS) (unsaturated lipid peroxidation byproducts indicative of oxidative stress). Interestingly, while SCD1 KO-induced reductions in lipid desaturation did not result in significant alterations to lipid peroxidation, it did cause worsened systemic inflammation and hyperglycemia in obese mice, indicative of a more severe diabetic phenotype. Importantly, aspartate aminotransferase (AST) and alanine aminotransferase (ALT), indicators of hepatocyte damage, were both significantly elevated ~2.7 and ~1.9-fold, respectively, in K_db_H_SCD1_ mice and ~3.8 and ~2.9-fold, respectively, in K_db_K_SCD1_ mice relative to lean controls, indicating profound obesity-induced hepatocellular stress worsened by SCD1 KO. The observed sarcopenia and hepatic remodeling are prognostic of severe metabolic stress affecting each of these essential lipid homeostasis regulatory organs (Chadt et al., 2000; Rohm et al., 2022).

### Skeletal muscle-specific SCD1 KO does not significantly affect obese mouse whole-body mass or composition

**Figure 4:**
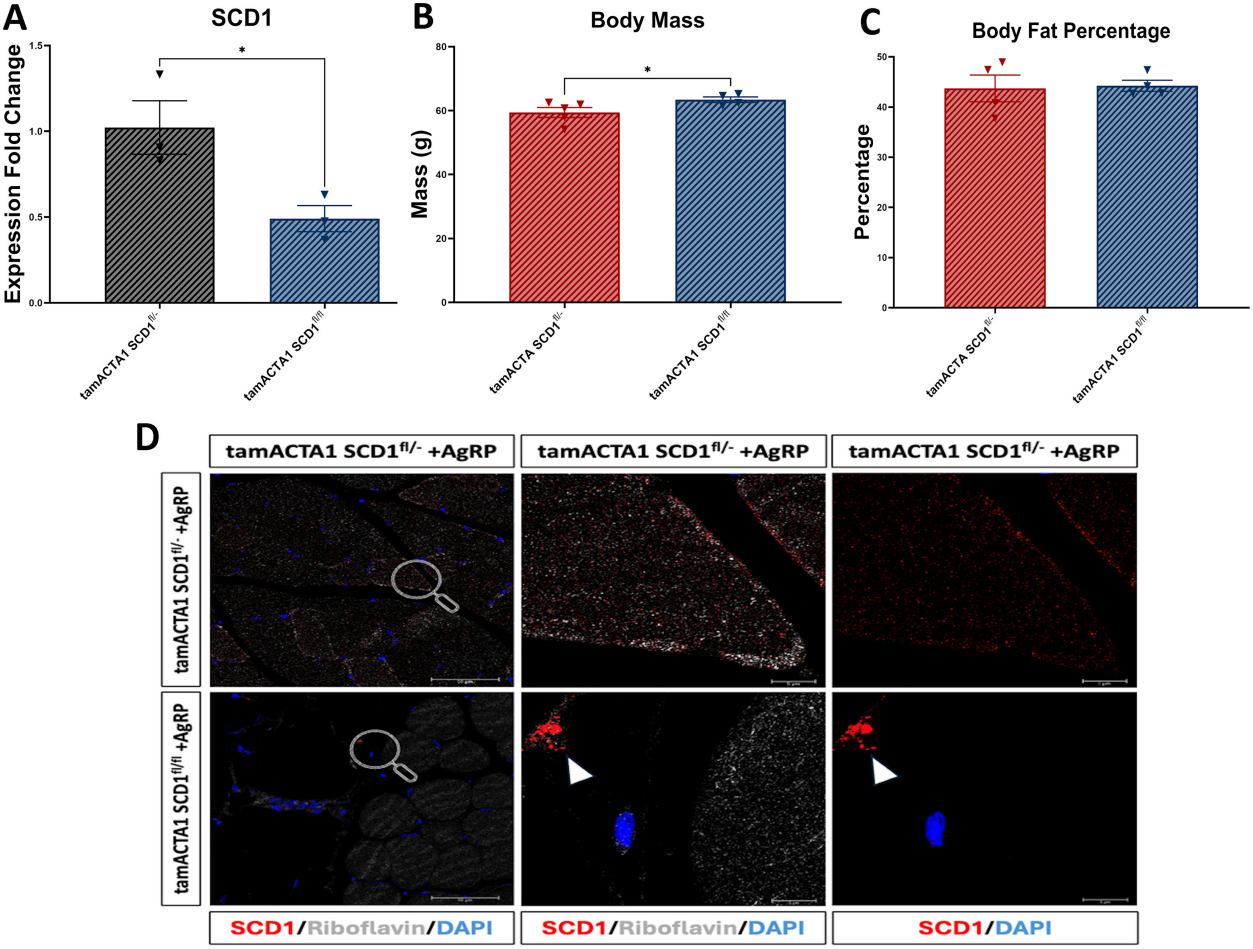
Tamoxifen-induced skeletal muscle-specific SCD1 KO results in modestly elevated AgRP AAV-induced whole-body weight gain and no significant effect on body composition. **(A)** RT-qPCR–determined gastrocnemius SCD1 mRNA levels (t-test, *n* = 3). **(B)** Mouse body masses (t-test, *n* ≥ 4) were used by Minispec Body Composition Analyzer to calculate **(C)** nuclear magnetic resonance (NMR)–determined body fat percentages (t-test, *n* = 4). **(D)** Anti-SCD1(red) staining of mouse gastrocnemius cross-sections was used to verify myofiber specificity of tamoxifen-induced KO relative to intramuscular adipocytes (white arrow) (left scale bar = 50 µm, middle and right scale bars = 5 µm). All data represented as mean ± SEM (*P < 0.05, **P < 0.01, ***P < 0.001, ****P < 0.0001).

As the role of SCD1-regulated intramyocellular lipid accumulation in the pathophysiology of obesity is unknown, we deployed a recently developed model from our labs that is mechanistically comparable to other leptin/LEPR antagonistic models (Sellers et al., 2026). Eight-week-old WT and striated myocyte-specific HSA-MCM (human alpha-skeletal actin (i.e., ACTA1) MerCreMer) mice on the SCD1^fl/±^ background were injected with a brain-targeted AAV to overexpress agouti-related peptide (AgRP) within the hypothalamus, triggering hyperphagia. They were allowed to age to 40 weeks to achieve weights consistent with other monogenic obesity models (e.g., db/db mice) (~60+ g), after which ACTA1cre mice were injected with tamoxifen to induce skeletal myofiber-specific SCD1 KO in fully floxed mice with established obesity (**Figure 4A**). Interestingly, SCD1 KO resulted in a significant 4.3 g increase to whole-body mass without affecting fat content (**Figures 4B, C**). To verify myofiber specificity of SCD1, we utilized immunofluorescence to illustrate its presence, via anti-SCD1+ red puncta shown against mitochondrial riboflavin autofluorescence, in tamACTA1 SCD1^fl/−^+AgRP gastrocnemius myofibers as well as its myofiber-specific deletion, with corresponding adipocyte preservation (white arrows), in SCD1^fl/fl^ muscle (**Figure 4D**).

### Loss of SCD1-mediated intramyocellular lipid storage capacity limits transcytosis-mediated FA uptake in obese mice

**Figure 5:**
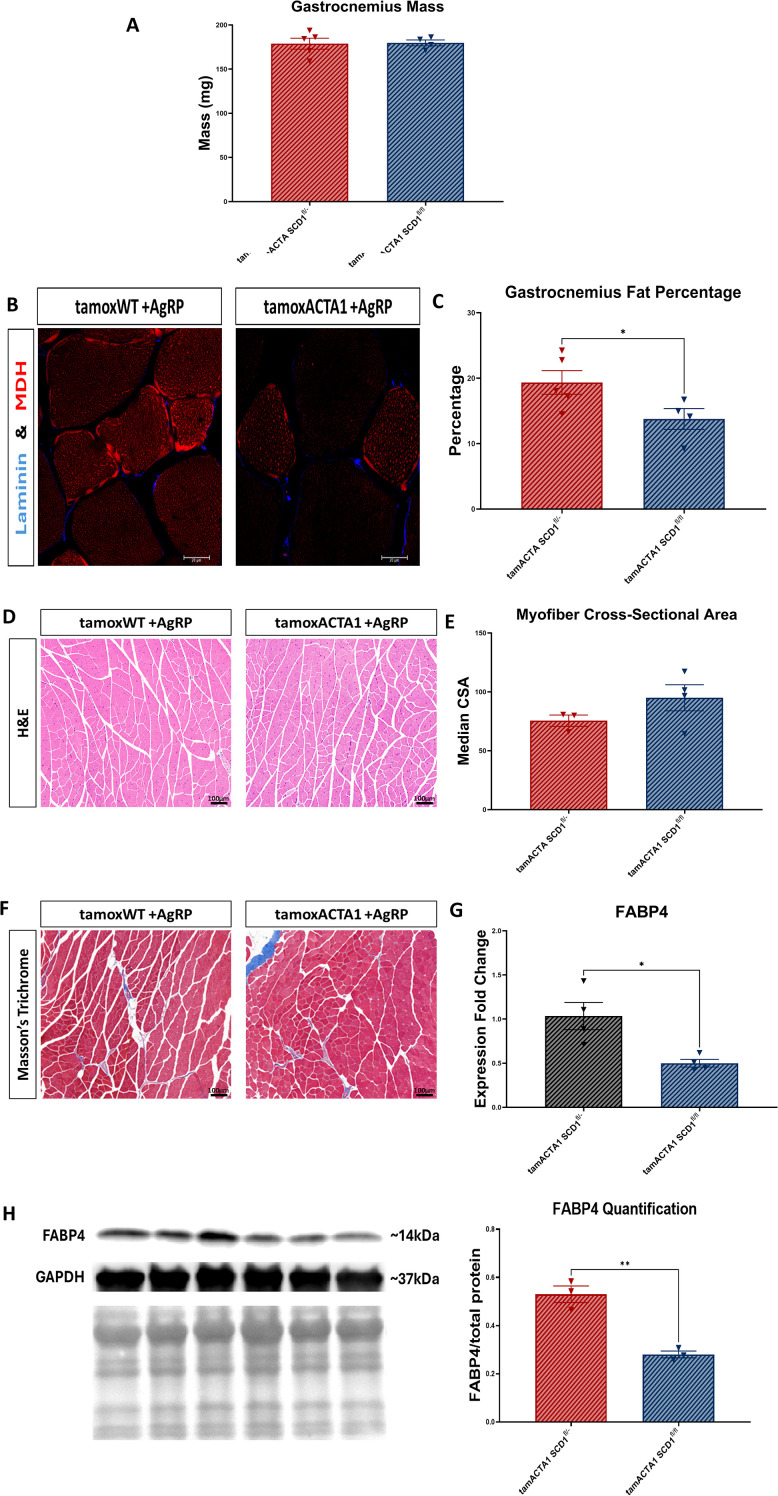
Loss of SCD1 expression in muscle prevents myosteatosis and limits expressions of FABP4. **(A)** Mouse gastrocnemius masses (t-test, *n* ≥ 4). **(B)** Representative Monodansylpentane (MDH, red) and anti-Laminin (blue) (scale bar = 20 µm) staining of mouse gastrocnemius cross-sections **(C)** nuclear magnetic resonance (NMR)–determined tissue fat percentages (t-test, *n* ≥ 4). **(D)** Representative H&E images of gastrocnemius cross-sections (scale bar = 100µm) and **(E)** corresponding myofiber cross-sectional area (CSA) quantification (t-test, *n* ≥ 3). **(F)** Representative Mason’s trichome (scale bar = 100 µm). **(G)** Gastrocnemius FABP4 mRNA levels (t-test, *n* = 4). **(H)** Western blot analysis of muscle FABP4 levels normalized to total protein (t-test, *n* = 3). All data represented as mean ± SEM (*P < 0.05, **P < 0.01, ***P < 0.001, ****P < 0.0001).

As shown in **Figure 5** and **Table 3**, induction of obesity in ACTA1 SCD1^fl/−^ mice resulted in significant loss of tibialis anterior (TA) muscle mass and a similar degree of steatosis seen in db/db mice (**Figure 2**). In contrast to global SCD1 deletion, induced SKM SCD1 KO preserved gastrocnemius mass while causing a robust 75% reduction in fat content (**Figures 5A–C**). We observed that SCD1 deletion resulted in no significant changes to myofiber morphology or cross-sectional area (CSA) (**Figures 5D–F**). Interestingly, using RT-qPCR, we found that SKM appeared to adapt to loss of lipid storage capacity via limiting endothelial transcytosis-mediating FA chaperone FABP4 expression by ~2-fold (**Figure 5G**). Changes in FABP4 gene expression correlated with a significant ~50% reduction in protein levels (**Figure 5H**). Furthermore, the expressions of other FA transporters (CD36, FABP3, and FATP1), lipid storage proteins (FITM2 and PLIN2), and insulin-dependent glucose transporter GLUT4 were not significantly changed, though perilipin 2 (PLIN2) displayed a trending decrease (**Supplementary Figure 1**). These results imply that loss of SKM SCD1 enzymatic activity restricts circulating FA uptake and therefore its SKM-derived disposal in obese mice.

**Table 3:** tamACTA1/SCD1fl/±+AgRP physiologic and metabolic phenotype data.

A.	Organ mass	
Genotype:	tamACTA1/SCD1^fl/−^+AgRP	tamACTA1/SCD1^fl/fl^+AgRP
Heart:	157.00 ± 7.0	157.95 ± 5.3
Soleus:	9.63 ± 0.40	10.08 ± 0.46
Tibialus anterior (TA)	80.900 ± 4.76	67.925 ± 3.31^*^
B.	Blood/plasma analysis	
Genotype:	tamACTA1/SCD1^fl/−^+AgRP	tamACTA1/SCD1^fl/fl^+AgRP
Insulin (ng/ml)	3.76 ± 1.41	2.52 ± 1.10
Leptin (pg/ml)	1520.5 ± 137.8	1480.8 ± 161.6
Triglyceride (mg/dl)	89.46 ± 16.8	82.76 ± 8.1
Cholesterol (mg/dl)	115.60 ± 4.5	143.96 ± 10.8^*^
NEFA (ng/ml)	0.8643 ± 0.024	0.8920 ± 0.036
Glucose (mg/dl)	213.00 ± 21.9	375.00 ± 70.7^0.051^
TBARS (µM)	0.3429 ± 0.054	0.3014 ± 0.031
HbA1c (%)	5.25 ± 0.27	6.48 ± 0.47*
TNFα (pg/ml)	50.621 ± 13.7	41.698 ± 10.9
AST (U/L)	59.591 ± 2.90	60.057 ± 2.62
ALT (pg/ml)	61.715 ± 0.38	63.960 ± 1.35^0.098^

Data was analyzed with Student’s t-tests and Mann-Whitney tests. All data represented as mean ± SEM (A) N = 4; (B) N ≥ 4, *P < 0.05, **P < 0.01, ***P < 0.001.

### Skeletal muscle SCD1 activity moderates hepatic lipid metabolism and resulting tissue content

**Figure 6:**
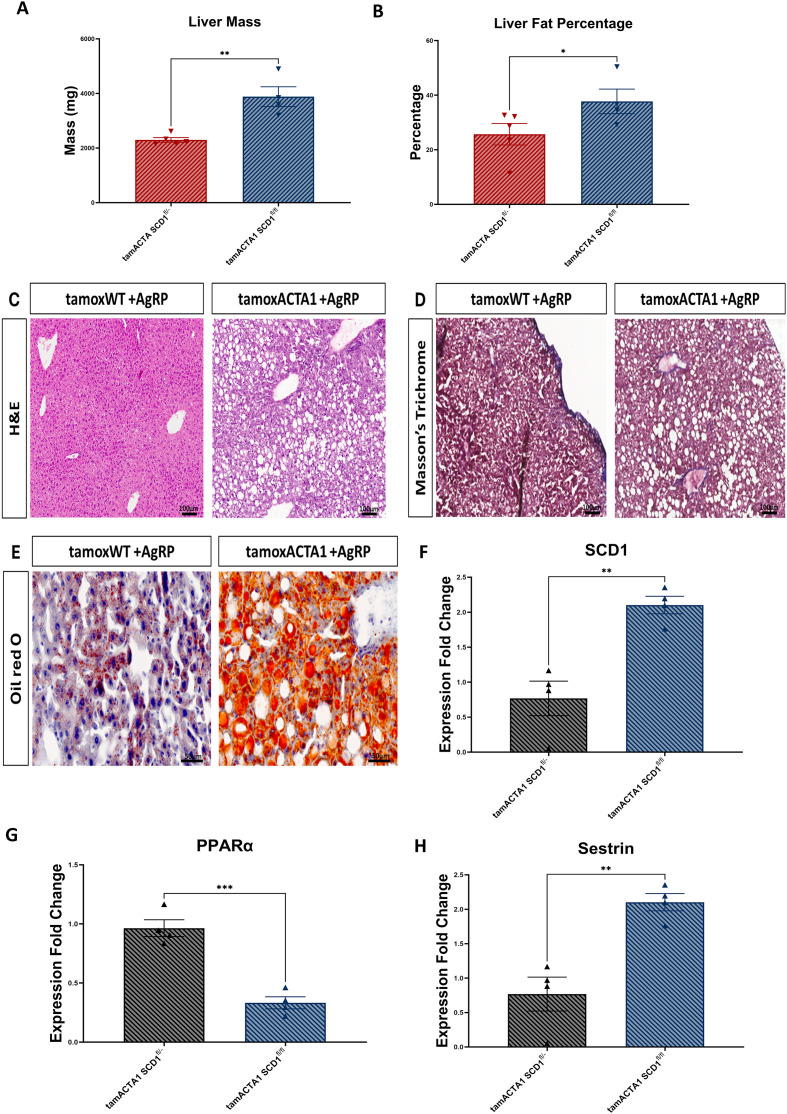
Myofiber SCD1 KO results in significantly elevated hepatomegaly and lipid accumulation. **(A)** Mouse liver masses (t-test, *n* ≥ 4) were used by Minispec Body Composition Analyzer to calculate **(B)** nuclear magnetic resonance (NMR)–determined tissue fat percentages (t-test, *n* ≥ 4). **(C)** Representative H&E (scale bar = 100 µm), **(D)** Mason’s trichome (scale bar = 100 µm), and **(E)** Oil Red O (ORO) (scale bar = 50 µm) staining of mouse liver cross-sections were performed to assess gross morphology and relative hepatocyte lipid content. **(F)** Liver SCD1, **(G)** PPARα, and **(H)** Sestrin mRNA levels (t-test, *n* = 4). All data represented as mean ± SEM (*P < 0.05, **P < 0.01, ***P < 0.001, ****P < 0.0001).

In contrast to the resolution of myosteatosis, SKM SCD1 KO greatly exacerbated obesity-induced hepatomegaly/steatosis-driven 43% and 34% increases in liver mass and fat content, respectively (**Figures 6A, B**). This pro-MAFLD effect is illustrated by the pervasive number of lipid-laden, ORO-positive hepatocytes evident in tamACTA1 SCD1^fl/fl^ + AgRP mice, which are not nearly as prevalent in their SCD1^fl/−^ counterparts (**Figures 6C–E**). In support of the observed MAFLD phenotype, gene expression analysis revealed that SKM SCD1 KO resulted in a compensatory twofold increase in liver SCD1 expression (**Figure 6F**). Additionally, elevated SCD1-mediated hepatosteatosis corresponded with a fourfold decrease in the master regulator of FA β-oxidation, PPARα, expression (**Figure 6G**). Similarly, downstream PPARα targets, PLIN2 and PLIN5 expressions are depressed. PPARα downregulation appears to be mediated, in part, by upregulation of its repressor carbohydrate response-element binding protein β (CHREBPβ), whose upregulation is in turn mediated by GLUT2-facilitated glucose import (**Supplementary Figure 2**). Next, we verified a ~2-fold upregulation of metabolic stress-responding Sestrin (aka SESN1) following induction of SKM SCD1 KO (**Figure 6H**). This data revealed the necessity of SCD1-mediated myocellular lipid uptake in the preservation of liver quality in obesity.

### Myofiber SCD1 KO worsens obesity-induced metabolic syndrome

Mirroring global KOs, SKM SCD1 deletion similarly resulted in significant elevations in plasma cholesterol and HbA1c (+20% and +19%, respectively) (**Table 3B**). Additionally, SCD1^fl/fl^ mice displayed a 43% increase in fasting blood glucose (*p* = 0.051). SKM SCD1 has been noted to facilitate the esterification, desaturation, and removal of circulating cholesterol and fatty acids, which if allowed to accumulate in hypercholesterolemia, can have cytotoxic effects on cells forced to store them in excess (i.e., hepatocytes) (Stamatikos and Paton, 2013). Myofiber SCD1 KO-driven reduced expression of key regulator of fatty acid uptake, FABP4 (**Figures 5F–H**), likely contributes to observed hypercholesterolemia and shift in liver lipid metabolism favoring storage at the expense of liver quality. These data indicate that muscle SCD1 is necessary for efficient glucose and lipid turnover and the prevention of metabolic syndrome-induced multi-organ pathophysiologic remodeling in obesity.

## Discussion

The deposition of ectopic lipids and the consequences thereof remain poorly understood components of obesity. We illustrate that SCD1-mediated lipid storage protects against the progression of obesity-induced hyperglycemia and T2D. Importantly, our study highlights the essential role of SKM myosteatosis in the partitioning of lipid burden away from the liver to preserve insulin sensitivity and protect against MAFLD in obesity. Furthermore, this protection may be mediated by SCD1’s ability to coordinate FABP4-regulated lipid uptake with intramyocellular storage. Collectively, these findings provide novel insight into SCD1-mediated myosteatosis as an important protective mechanism in the preservation of insulin signaling, underscoring the potential of SKM-targeted SCD1 agonism in the treatment of obesity.

Obesity is a state of chronic nutrient excess whereby supply supersedes both metabolic demand and rate of disposal, leading to accumulation in the form of lipid storage, initially in extent adipocytes driving hypertrophy followed by hyperplasia, both of which contribute to expansion of visceral and subcutaneous fat depots. Additionally, as nutrient intake surpasses critical adipose storage thresholds, spillover (i.e., steatosis) into other metabolic organs, such as infiltrating extra-/intercellular adipocytes and elevated intracellular lipid content, occurs. Progressively, excessive intracellular lipid levels lead to the cytotoxic accumulation of bioactive lipid intermediaries, which are primary instigators of mitochondrial and ER stress, elevated reactive oxygen species (ROS), and impaired insulin signaling. In this way, obesity-driven steatosis acts as a “one-two punch” against SKM and liver quality, which contributes to local and systemic low-grade inflammation and precipitates the progression of insulin resistance, metabolic syndrome, T2D, and cardiovascular disease (Fabbrini et al., 2010; Stepp, 2010; Britton and Fox, 2011; Snel et al., 2012; Heymsfield and Wadden, 2017; Prasun et al., 2021; Jin et al., 2023; Padgett et al., 2023; Janssen, 2024; McNaught, 2026). In this context, promoting a greater understanding of the processes underlying steatosis may open new avenues for the development of effective pharmacotherapies aimed at treating obesity-associated disease.

Indeed, our lab, and others, have previously reported on the efficacy of reversing hepatosteatosis as a means of improving insulin sensitivity and metabolism (Brunner et al., 2019; Larion et al., 2022; Beygi et al., 2024). Similarly, it is well documented that exercise-mediated improvements to SKM quality provide systemic metabolic improvements in obese individuals, even in the absence of weight loss (Gaesser et al., 2011; Strasser, 2013; Petri Wiklund et al., 2014; Wiklund et al., 2014). In fact, we have found that, in the absence of exercise, prevention of obesity-induced sarcopenia via MSTN KO protects cardiovascular function in db/db mice (Qiu et al., 2014; Butcher et al., 2018). Furthermore, we observed that preservation of muscle mass coincided with reductions in myosteatosis, leading us to identify SCD1 as a potential mediator of this phenomenon (data not shown). This notion is further validated by a wealth of human, animal, and cell data providing great mechanistic insight into SCD1’s function in catalyzing the addition of a *cis* double bond in the Δ9 position of fatty acyl-CoAs, with preferred substrates being palmitoyl-CoA and stearoyl-CoA, which it converts to palmitoleoyl-CoA (16:1) and oleoyl-CoA (18:1), respectively. In this way, SCD1 acts as the rate-limiting step in MUFA synthesis, producing essential components for TG and cholesterol ester synthesis, the most sterically stable/efficient neutral FAs for lipid droplet storage (Enoch et al., 1976; Kasturi and Joshi, 1982; Miyazaki et al., 2001). Thus, in the absence of SCD1 activity, lipid storage is greatly impaired, leading to reduced adipocyte expansion, steatosis, and weight gain in obesity (Ntambi et al., 2002). We confirmed these conclusions in our db/SCD1 double-KO mice. However, others have reported that global SCD1 KO on an atherogenic Western diet model or hepatic SCD1 KO on a high-carbohydrate diet model promotes systemic inflammation-driven increases in lesion size and oleate deficiency-mediated ER stress, respectively (MacDonald et al., 2009; Aljohani et al., 2019). Similarly, we found that global db/SCD1 double-KO mice have greatly elevated plasma TNF, AST, and ALT concentrations; evident pathological liver remodeling; worsened sarcopenia; and severe diabetic symptoms. In this way, the diverse and selective reporting of context-dependent and often contradictory effects of SCD1 antagonism necessitates careful attention to the obesity model used and tissue/cell specificity of SCD1 targeting.

Based on previous reports revealing SCD1 RNA and protein to be upregulated in both obese and endurance-trained athlete SKM, we hypothesized that SKM SCD1-mediated intramyocellular lipid accumulation is a double-edged sword, beneficial in ensuring adequate nutrient supply under conditions of intense energy demand but detrimental in promoting lipotoxicity in the context of obesity (Schenk and Horowitz, 2007). Additionally, as leptin is a major regulator of liver, adipose, and SKM lipid metabolism (via its inhibition of SCD1 and activation of FAO) as well as a contributor to vascular tone (Flowers et al., 2007; Sampath et al., 2009; Belin de Chantemele et al., 2011), we reasoned that by eliciting hyperphagia via brain-specific AgRP overexpression without directly affecting peripheral leptin signaling, we could better model human obesity and eliminate confounding variables (Sellers et al., 2026). We tested these theories via the creation of an inducible SKM-specific SCD1 KO within our AgRP obesity model. In contrast to our expectations, we found that SKM SCD1 KO led to a significantly worsened metabolic syndrome phenotype characterized by hyperglycemia and hypercholesterolemia. Excitingly, we identified the differential expression of the novel SCD1-FABP4 lipid flux regulatory axis in SKM as a putative driver of metabolic dysfunction in tamoxifen-treated ACTA1 SCD1^fl/fl^ obese mice. These findings contradict long-standing dogma painting SCD1 and myosteatosis as “bad actors” propagating obesity-induced metabolic dysfunction, instead revealing them to be a protective counterbalance against MAFLD and insulin resistance.

Limitations of the current study lie primarily in the next generation of questions raised by these findings. A key open question is whether the redistribution of fat that occurs with deletion of SKM SCD1 reflects simply a subtractive effect in which the inability to dispose of lipids in one source simply overburdens another or perhaps more intriguingly, whether lipid rebalance reflects the disruption of a network of inter-organ communication in which the lipidemic status of muscle changes the release of factors such as myokines that alter the behavior of remote organs. Another open question is what stimulates the expression and activity of SCD1 in insulin resistant muscle. If insulin signaling is lost, then what drives lipid accumulation? Is there selective insulin resistance such that lost glucose uptake in response to insulin allows the muscle to store other substrates to maintain muscle health. It is hope that these findings will inspire deeper exploration into more detailed mechanisms by which muscle lipid homeostasis contributes to overall energy balance in obesity.

**Figure 7:**
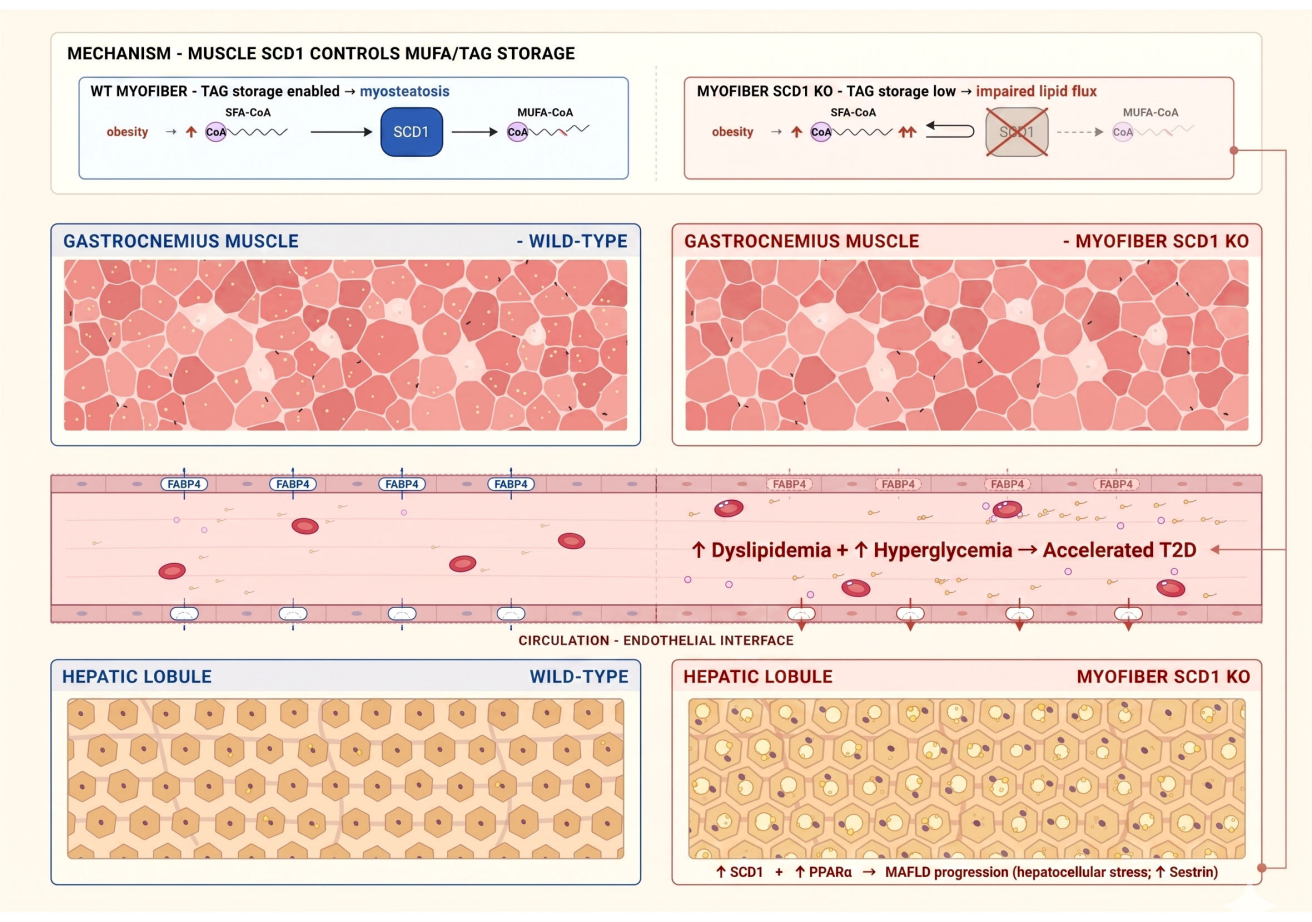
Graphical abstract of the role of SCD1 in skeletal muscle in the context of obesity.

In conclusion, our study found that (1) SCD1-mediated lipid storage is critical to preserve metabolic homeostasis in obesity (2); myocellular SCD1 expression limits obesity-driven hyperglycemia and hypercholesterolemia, protecting against MAFLD; and (3) loss of myofiber SCD1 impairs FABP4-mediated FA transcytosis, redirecting circulating lipids to be stored in the liver (**Figure 7**). These findings reveal the intrinsic nature of SCD1 in SKM-liver lipid crosstalk, definitively identifying it as a primary regulator of myosteatosis, an identity that could potentially be pharmacologically leveraged in the treatment of obesity.

### Clinical implications

Myosteatosis is a well-documented aspect of muscle biology in obesity. In clinical studies, the extent of myosteatosis correlates with the severity of insulin resistance or metabolic syndromes but the question of whether this ectopic lipid accumulation is casual, correlative, or compensatory has remained an open question. A key concept promoted by this work is that myosteatosis may be adaptation to excess lipid that at least in some degree fuels muscle when carbohydrate stores are lacking and potentially protects organs from the liver from even worser steatosis which has a greater impact on the progression of metabolic disease.

## Data Availability

The original contributions presented in the study are included in the article/**Supplementary Material**. Further inquiries can be directed to the corresponding author.
