# Supplementary material for: Myofiber stearoyl-CoA desaturase 1 moderates MAFLD and metabolic syndrome in obese mice
Source: Front Physiol. 2026 Aug 3;17:1826756. doi: 10.3389/fphys.2026.1826756 (PMC13478006; doi:10.3389/fphys.2026.1826756)
Supplement: Supplementary file 1 [file DataSheet1.docx]

**Supplementary Table 1.**

| **Mouse primer sequences for genotyping** | | |
| --- | --- | --- |
| db | Fwd | 5’- CCC AAC AGT CCA TAC AAT ATT AGA AGA TTT TTA CAT TTT GA -3’ |
| MUT | Rev | 5’- GTC CAA ACT GAA CTA CAT CAA ACC TAC -3’ |
| SCD1 | Fwd | 5’- GGG TGA GCA TGG TGC TCA GTC CCT -3’ |
| WT | Rev | 5’- CAC ACC ATA TCT GTC CCC GAC AAA TGT C -3’ |
| SCD1 | Fwd | 5’- ATA GCA GGC ATG CTG GGG AT -3’ |
| MUT | Rev | 5’- CAC ACC ATA TCT GTC CCC GAC AAA TGT C -3’ |
| SCD1^fl/±^ | Fwd | 5’- TGG AGC CAC AGA ACT TAC AA -3’ |
| WT | Rev | 5’- GCC CAG CAC AGC CAA TAG -3’ |
| SCD1^fl/±^ | Fwd | 5’- TCC AGG TAA GAA GCG AGT GC -3’ |
| MUT | Rev | 5’- TGA GGC GGA AAG AAC CAG -3’ |
| ACTA1-MCM | Fwd | 5’- AGT GGC CTC TTC CAG AAA TG -3’ |
| internal+ctl | Rev | 5’- TGC GAC TGT GTC TGA TTT CC -3’ |
| ACTA1- MCM | Fwd | 5’- CAG GTA GGG CAG GAG TTG G -3’ |
| MUT | Rev | 5’- TTT GCC CCC TCC ATA TAA CA -3’ |
| **Mouse primer sequences for qPCR** | | |
| SCD1 | Fwd | 5’- TTC TTA CAC GAC CAC CAC CA -3’ |
|  | Rev | 5’- GCA GGA GGG AAC CAG TAT GA -3’ |
| FABP4 | Fwd | 5’- TTC CTT CAA ACT GGG CGT GG -3’ |
|  | Rev | 5’- GCC TTT CAT AAC ACA TTC CAC C -3’ |
| PPARα | Fwd | 5’- CGA ACA TTG GTG TTC GCA G -3’ |
|  | Rev | 5’- GTG ATG ACA ACG TCT TGT T -3’ |
| SESN1 | Fwd | 5’- CGC AGC CGT CCC TTC G -3’ |
|  | Rev | 5’- GAT TCT GAT TCC AAG TTC CTC GTC -3’ |
| GAPDH | Fwd | 5’- CAT CAC TGC CAC CCA GAA GAC TG -3’ |
|  | Rev | 5’- ATG CAG TGA GCT TCC CGT TCA G -3’ |
| 18s | Fwd | 5’- GTA ACC CGT TGA ACC CCA TT -3’ |
|  | Rev | 5’- CCA TCC AAT CGG TAG TAG CG -3’ |
| **Western blot antibodies** | | |
| Anti-FABP4 antibody [EPR3579] (ab92501) (Abcam) | | |
| GAPDH (14C10) Rabbit Monoclonal Antibody #2118 (Cell Signaling) | | |
| Anti-rabbit IgG, HRP-linked Antibody #7074 (Cell Signaling) | | |

**Supplementary Figure 1.**

**Supplementary Figure 1. RT-qPCR reveals myofiber SCD1 KO results in unsignificant changes to expressions of other lipid transporters (CD36,FABP4, and FATP1), trending decreases in lipid storage genes (FITM2 and PLIN2), and unsignificant changes to insulin-regulated GLUT4.**

**Supplementary Figure 2.**

**Supplementary Figure 2. RT-qPCR shows downregulation of hepatic** **PPARα results in significant reductions in expressions of downstream target lipid droplet-associated PLIN2 and PLIN5; furthermore, elevated GLUT2-mediated glucose import likely contributes to CHEBPβ-directed PPARα repression.**
